# Relationship between Self-Administered Cues and Rehabilitation Outcomes in Individuals with Aphasia: Understanding Individual Responsiveness to a Technology-Based Rehabilitation Program

**DOI:** 10.3389/fnhum.2017.00007

**Published:** 2017-02-01

**Authors:** Carrie A. Des Roches, Annette Mitko, Swathi Kiran

**Affiliations:** ^1^Aphasia Research Laboratory, Department of Speech, Language, and Hearing Sciences, Sargent College, Boston UniversityBoston, MA, USA; ^2^Department of Communication Sciences and Disorders, MGH Institute of Health ProfessionsBoston, MA, USA

**Keywords:** aphasia, iPad-based rehabilitation, Constant Therapy, treatment, self-administered cues, individualized rehabilitation

## Abstract

An advantage of rehabilitation administered on computers or tablets is that the tasks can be self-administered and the cueing required to complete the tasks can be monitored. Though there are many types of cueing, few studies have examined how participants’ response to rehabilitation is influenced by self-administered cueing, which is cueing that is always available but the individual decides when and which cue to administer. In this study, participants received a tablet-based rehabilitation where the tasks were selfpaced and remotely monitored by a clinician. The results of the effectiveness of this study were published previously (Des Roches et al., 2015). The current study looks at the effect of cues on accuracy and rehabilitation outcomes. Fifty-one individuals with aphasia completed a 10-week program using Constant Therapy on an iPad targeted at improving language and cognitive deficits. Three questions were examined. The first examined the effect of cues on accuracy collapsed across time. Results showed a trend where the greater the cue use, the lower the accuracy, although some participants showed the opposite effect. This analysis divided participants into profiles based on cue use and accuracy. The second question examined how each profile differed in percent cue use and on standardized measures at baseline. Results showed that the four profiles were significantly different in frequency of cues and scores on WAB-R, CLQT, BNT, and ASHA-FACS, indicating that participants with lower scores on the standardized tests used a higher percentage of cues, which were not beneficial, while participants with higher scores on the standardized tests used a lower frequency of cues, which were beneficial. The third question examined how the relationship between cues and accuracy was affected by the course of treatment. Results showed that both more and less severe participants showed a decrease in cue use and an increase in accuracy over time, though more severe participants continued to used a greater number of cues. It is possible that self-administered cues help some individuals to access information that is otherwise inaccessible, even if there is not an immediate effect. Ultimately, the results demonstrate the need for individually modifying the levels of assistance during rehabilitation. time, though more severe participants continued to used a greater number of cues. It is possible that self-administered cues help some individuals to access information that is otherwise inaccessible, even if there is not an immediate effect. Ultimately, the results demonstrate the need for individually modifying the levels of assistance during rehabilitation.

## Introduction

Each year nearly 800,000 individuals suffer a stroke (Winstein et al., 2016) and roughly 1.3 million individuals suffer from brain injury (Corrigan et al., 2010). The language and cognitive deficits that result from these injuries can manifest as a chronic disability for these individuals and require long-term rehabilitation. Recent technological advances make tablet-based rehabilitation a feasible option for these individuals (Holland, 2014; Hoover and Carney, 2014; Kiran et al., 2014; Kurland, 2014; Kurland et al., 2014; Ramsberger and Messamer, 2014; Szabo and Dittelman, 2014; Des Roches et al., 2015; White et al., 2015; Lee and Cherney, 2016; Routhier et al., 2016; Zheng et al., 2016). However, despite significant advances in aphasia rehabilitation approaches, it is still difficult to predict and explain which individuals benefit from treatment and which individuals do not due to the vast differences in the way rehabilitation is provided to patients (Best and Nickels, 2000; Carlomagno et al., 2001; Lazar et al., 2008). Another important factor contributing to our lack of complete understanding of who benefits from treatment and who does not is the inherent heterogeneity of individual patients in terms of their profile (e.g., age, months post stroke, severity of language impairment, levels of motivation, etc.) that can impact performance (Goodglass et al., 1966; Hanson et al., 1989; Schwartz and Brecher, 2000; Hilari et al., 2003; Pedersen et al., 2003; Murray, 2012; Hachioui et al., 2014).

**FIGURE 1:**
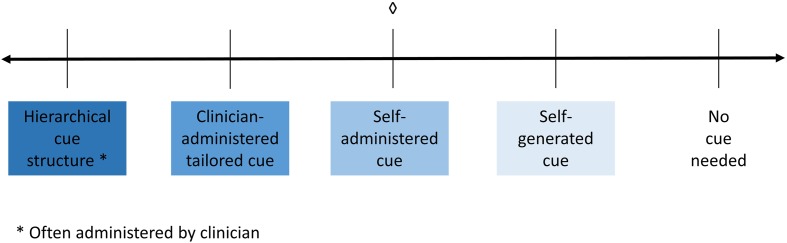
**Schematic demonstrating the spectrum of cueing, where hierarchical cueing (provided by a clinician) falls at on the left side of the spectrum**. Following that lies clinician administered cueing where the clinician decides when and which cues to administer. In the center is self-administered cueing, which is when cues are available (provided by a software program) and the patient chooses when and which cues to administer to themselves. Next is self-generated cueing where the patient generates the cue for themselves. Finally, on the right side of the spectrum is where the patient does not need a cue. ♢, what the current study is examining.

A third and equally important factor that might affect performance or improvement in rehabilitation is the level and type of cueing (or prompts) that an individual may require to complete a given task. Depending on the tools available to them, clinicians have to make a decision about what type of cueing to provide to their patients. The amount of assistance provided by the clinician, the level of independence and the structure that the cue types provide can range widely on a continuum, with most assistance to least assistance, as shown in the schematic in **Figure 1**. At the left end of the continuum is a hierarchical type of cueing, where the clinician sets up and administers a predetermined hierarchy of differing cues (i.e., phonemic cues, orthographic cues, etc.); there is minimal patient independence. Next on the continuum is clinician-administered type of cueing, where the clinician determines what cues to provide and when to provide them but also takes participant input. Next, self-administered cues are cues that are always available to participants (e.g., through a software program), but they need to determine what cues they would use and when to use them with or without clinician assistance. Almost to the far right of the continuum are self-generated types of cues, where participants are trained to generate cues for themselves that can be implemented independently. Finally, at the right end of the continuum is no cueing, which is when the participant can perform tasks independent of any cues.

These different cues have been examined in several studies in terms of their impact on treatment outcomes. Following **Figure 1**, hierarchical cueing structure has been well studied as a treatment method and has been shown to be effective (Wambaugh et al., 2001; Linebaugh et al., 2005; Cameron et al., 2006; Choe and Stanton, 2011; Conroy and Scowcroft, 2012). For example, Cameron et al. (2006) examined a treatment with five individuals with aphasia using a combined semantic and phonological cueing hierarchy applied to information units that were missing from story retelling and found that four of the five participants showed improvements on retrieval of trained items, while the remaining participant showed an improvement on another discourse measure. Next, several treatment studies involve clinician-administered cues, which have been also been found to be effective. One study examined semantic cueing as part of a naming therapy (Lowell et al., 1995), where cueing was structured and administered by a clinician with the help of a semantic feature analysis diagram. The authors found the therapy to be effective for two of three patients. Another study examined a clinician-administered cueing treatment which worked on cueing verbs to elicit sentence production, where verbs were presented along with six hierarchical levels of wh-cues (questions introduced by a wh-word such as who, where, why, etc.) that patients could progress through (Loverso et al., 1987). This type of verb-cueing was found to be effective. Self-administered cueing has previously been studied in several studies. One such study examined the effectiveness of a computer-delivered self-administered cueing program, MossTalk in either a clinician-guided or a partially self guided condition (Fink et al., 2002) and the authors found that both treatment conditions were effective. Another study examined the effectiveness of self-administered cues using MossTalk at home in four individuals, and found it to be effective for acquisition and maintenance of trained items (Ramsberger and Messamer, 2014). Yet another study examined the effectiveness of a computer-based therapy program, called Multicue, which allowed patients to select which cue(s) they wanted to use to help them name a picture (Doesborgh et al., 2004). The authors found that patients who used Multicue improved on confrontation naming but did not improve in other measures of verbal communication. Finally, one study examined the use of personalized, self-generated cues to work on naming in a single patient (Freed et al., 2004), which was based on several other studies examining personalized cueing for learning either word-symbol associations or dog breeds (as cited in Marshall and Freed, 2006). Freed et al. (2004) found improvements in naming in both the personalized cueing method and in a typical phonemic cueing method of treatment.

To summarize, the findings of these previous studies show that different types of cueing can be effective for improving word retrieval skills for different types of patients, but these gains are constrained by the individual severity of the patients. The current study focuses only on self-administered cues delivered through a software program. Self-administered cues provide an important insight into patients’ awareness of their inability to complete a language task independently. It can be surmised that there are several important internal steps involved when one self-administers cues. First, the individual needs to realize they do not know or cannot produce the correct answer and then recognize that they need help in order to produce the correct answer. Next, the individual self-administers the cue and finally uses the cue to help them produce the correct answer. However, the internal processing mechanisms of self-administered cues are not completely understood (see Tompkins et al., 2006 for a similar discussion) and begs systematic examination.

The current paper focuses on three aspects of self-administered cue use; the effect of cue use on performance, individual variability, and the effect of cue use over time on performance. A recent study (Cherney et al., 2014) examined individual patient variability and degree of cue use in a different context, cues for scripts (AphasiaScripts). Cherney et al. (2014) found no significant differences by severity, whether the cues were provided more or less frequently. However, there was a greater amount of change in the higher cueing condition than the lower cueing condition for more severe individuals with aphasia. Taken together with the studies discussed above, this study provides converging evidence that individuals with severe aphasia utilize cues differently than individuals with less severe aphasia, and this difference may also be impacted by the degree of cue use. Another study has examined the effect of cue use over time, in the context of a hierarchical cueing protocol (Boyle and Coelho, 1995). Results showed that when the clinician directed the hierarchical cues, the participant was able to internalize the structure of the cues and self-generate the cues over the course of the therapy, needing fewer cues later in therapy. This study hints at the potentially important influence that cues initiated by the individual may have in shaping the language behavior over the course of treatment.

Importantly, computer-delivered self-administered cues provide a unique yet untested opportunity to ascertain the benefit of self-administered cues as they can be systematically logged and analyzed. The present study, thus, examined the influence of such computer-delivered self-administered cues on improving language performance and how cue use changed as a function of treatment. In a previous study, 51 individuals with aphasia of varied severity profiles were studied while utilizing the Constant Therapy software application (Constant Therapy, Inc., Newton, MA, USA) over a 10 week treatment program (Des Roches et al., 2015), where a range of tasks were assigned to participants based on participants’ language and cognitive severity. Results from accuracy and latency in the treatment, as well as on standardized outcome measures, demonstrated that the treatment was effective. Many of the treatment tasks in Constant Therapy have a cue feature (described in greater detail below) allowing a closer examination of the way participants interact with self-administered cues and how utilization of cues may influence treatment outcome. Based on the evidence discussed from previous studies, it is not clear how cue use varies with individual patient severity, how cue use changes over time, or how both of these affect performance.

Therefore, the aim of the current study was to investigate the relationship between self-administered cues, participant severity and corresponding accuracy on the treatment tasks in these 51 participants. The following questions were examined in this study:

1 (a) What is the relationship between accuracy and cue use for individual participants, and (b) are there certain profiles of cue use that are common across participants? It was predicted that most participants would demonstrate a relationship where increased cue use would result in higher accuracy as they use cues to aid their performance.
2 What is the relationship between participants’ severity profiles and self-administered cue use? It was hypothesized that participants would show one of four possible outcomes: (1) cue use that is beneficial to their performance, (2) cue use that is not beneficial to their performance, (3) a lack of cue use but poor performance, or (4) a lack of cue use and good performance. It was expected that more and less severe participants would show different outcomes.
3 How does self-administered cue use influence improvement in accuracy as a function of treatment? It was hypothesized that the more and less severe participants would show one of four possible outcomes: (1) cue use that is beneficial to their performance in the treatment, (2) cue use that is not beneficial to their performance in the treatment, (3) a lack of cue use but poor performance in the treatment, or (4) a lack of cue use and good performance in the treatment. It was again expected that more and less severe participants would show different outcomes.

## Materials and Methods

### Participants

Fifty-one individuals (20 female) were recruited and completed the 10 weeks treatment program described in the previous study (Des Roches et al., 2015). Written informed consent was obtained for all participants, in accordance with policies set forth by the Boston University Institutional Review Board. All participants suffered either a stroke or a traumatic brain injury, ranging in months post onset (MPO) from one to 359 months (*M* = 59.6, *SD* = 69.5). Participants ranged in age from 38 to 87 years (*M* = 64.2, *SD* = 10.7). Refer to **Table 1** for all participants’ age and MPO.

**Table 1:** Participant demographic and severity information, including age, MPO, cause of injury, and standardized test scores: WAB-R Aphasia Quotient, CLQT Composite Severity, BNT, PAPT, and ASHA-FACS Communication Independence Mean and Qualitative Dimension Mean scores.

				WAB-R	CLQT			ASHA-FACS		Severity Bin (more or less severe)	Frequency of cue use (%)
ID	Age	MPO	Cause	AQ	CS	BNT	PAPT	CI Mean	QD Mean		
1	75	63	Stroke	64.4	65.0%	46.7%	82.7%	DNT	DNT	More severe	16.3
2	67	60	Stroke	31.3	35.0%	0.0%	80.8%	4.8	3.5	More severe	56.0
3	71	65	TBI	27.8	25.0%	0.0%	59.6%	4.2	2.2	More severe	42.2
4	56	141	Stroke	93.2	90.0%	75.0%	96.2%	6.0	4.6	Less severe	37.9
5	71	24	Stroke	42.4	50.0%	0.0%	65.4%	DNT	DNT	More severe	41.2
6	72	22	Stroke	77.9	85.0%	85.0%	92.3%	6.1	4.2	Less severe	21.0
7	58	75	Stroke	80	55.0%	56.7%	96.2%	6.0	4.0	Less severe	7.0
8	75	24	Stroke	68.1	60.0%	16.7%	82.7%	6.3	4.2	More severe	81.0
9	38	16	Stroke	97.6	95.0%	90.0%	96.1%	6.7	4.8	Less severe	15.0
10	74	22	Stroke	DNT	DNT	DNT	DNT	DNT	DNT	More severe	12.1
11	76	177	TBI	65.9	75.0%	11.7%	78.8%	DNT	DNT	More severe	40.9
12	47	44	Stroke	96.3	90.0%	91.7%	98.1%	6.0	4.0	Less severe	15.6
13	68	87	Stroke	70.5	75.0%	45.0%	90.4%	6.2	4.3	More severe	24.7
14	50	33	Stroke	93.9	100.0%	98.3%	98.1%	7.0	4.6	Less severe	27.2
15	46	60	TBI	81.4	70.0%	15.0%	80.8%	4.2	3.4	Less severe	45.6
16	71	78	Stroke	12	30.0%	0.0%	67.3%	3.8	2.4	More severe	65.7
17	66	14	Stroke	60.2	25.0%	0.0%	63.5%	3.2	2.8	More severe	76.9
18	71	46	Stroke	44.5	70.0%	0.0%	84.6%	6.3	4.4	More severe	45.4
19	87	13	Stroke	88.7	65.0%	58.3%	DNT	5.6	4.3	Less severe	60.4
20	68	23	Stroke	59	45.0%	13.3%	76.9%	4.4	2.6	More severe	82.3
21	72	1	Stroke	11.5	60.0%	5.0%	82.7%	5.2	3.9	More severe	35.1
22	74	12	Stroke	67.6	65.0%	91.7%	92.3%	6.3	3.3	More severe	32.3
23	53	32	Stroke	91	75.0%	51.7%	94.2%	6.7	4.0	Less severe	29.1
24	58	109	Stroke	75.7	85.0%	86.7%	98.1%	6.8	3.7	Less severe	58.6
25	50	178	Stroke	59.3	50.0%	55.0%	90.4%	DNT	DNT	More severe	53.0
26	70	14	Stroke	15.6	60.0%	0.0%	73.1%	4.0	2.8	More severe	80.7
27	75	141	Stroke	93.4	55.0%	80.0%	88.5%	6.5	4.4	Less severe	30.0
28	52	27	Stroke	90.2	65.0%	43.3%	78.8%	5.8	4.1	Less severe	54.7
29	68	21	Stroke	95	95.0%	90.0%	96.2%	6.7	4.8	Less severe	18.3
30	68	22	Stroke	44.1	25.0%	1.7%	21.2%	3.6	2.3	More severe	96.5
31	74	29	Stroke	93.7	70.0%	93.3%	82.7%	6.2	3.6	Less severe	75.8
32	56	2	Stroke	97.2	85.0%	83.3%	92.3%	6.9	4.9	Less severe	17.7
33	74	15	Stroke	49.7	55.0%	28.3%	75.0%	3.9	2.5	More severe	61.7
34	38	54	Stroke	77.7	85.0%	55.0%	96.2%	6.3	4.2	Less severe	56.3
35	59	72	Stroke	98.9	100.0%	98.3%	98.1%	6.9	4.8	Less severe	16.6
36	83	41	Stroke	90.7	95.0%	90.0%	96.2%	6.2	4.3	Less severe	69.7
37	65	29	Stroke	27.9	55.0%	0.0%	84.6%	5.6	4.3	More severe	66.6
38	64	88	TBI	77.9	90.0%	60.0%	90.4%	6.4	3.9	Less severe	25.9
39	58	359	TBI	83.2	85.0%	25.0%	84.6%	6.4	4.8	Less severe	29.2
40	67	94	Stroke	73.7	50.0%	58.3%	96.2%	5.4	3.6	More severe	78.3
41	55	11	Stroke	71.3	55.0%	30.0%	78.8%	4.9	4.3	More severe	72.2
42	53	285	Stroke	93.9	100.0%	98.3%	98.1%	6.8	4.3	Less severe	23.9
43	66	31	Stroke	89.9	90.0%	73.3%	86.5%	6.8	4.6	Less severe	54.2
44	67	4	Stroke	99.9	95.0%	98.3%	96.2%	6.9	4.9	Less severe	44.0
45	66	18	Stroke	20.8	65.0%	0.0%	84.6%	3.9	2.4	More severe	98.7
46	61	54	Stroke	91.2	95.0%	95.0%	96.2%	6.5	4.4	Less severe	27.2
47	54	8	Stroke	21.6	25.0%	0.0%	46.2%	3.8	2.8	More severe	59.3
48	66	129	Stroke	48.7	70.0%	6.7%	90.4%	5.1	3.6	More severe	63.3
49	79	6	Stroke	DNT	DNT	DNT	DNT	DNT	DNT	More severe	50.9
50	60	46	Stroke	93.7	40.0%	93.3%	90.4%	5.2	3.9	Less severe	52.1
51	64	23	Stroke	75.9	85.0%	41.7%	98.1%	DNT	DNT	Less severe	51.3
Avg.	64.2	59.6	46 stroke,	68.9	68.0%	47.7%	84.7%	5.6	3.8	25 more severe,	47.0
*SD*	10.7	69.5	5 TBI	26.6	22.3%	37.4%	14.9%	1.1	0.8	26 less severe	23.4

*MPO, Months Post Onset; WAB-R AQ, Revised – Western Aphasia Battery Aphasia Quotient; CLQT CS, Cognitive Linguistic Quick Test Composite Severity; BNT, Boston Naming Test; PAPT, Pyramids and Palm Trees; ASHA-FACS, American Speech-Language-Hearing Association – Functional Assessment of Communication Skills for Adults; CI, Communication Independence; QD, Qualitative Dimension; DNT, Did Not Test; TBI, Traumatic Brain Injury. Two participants did not complete any testing due to inability and unwillingness to complete tests*.

Before beginning the treatment program, participants were administered the *Revised – Western Aphasia Battery* (WAB-R, Kertesz, 2007), which was used to determine the type and level of aphasia severity, the *Cognitive Linguistic Quick Test* (CLQT, Helm-Estabrooks, 2001), which was used to determine the relative contribution of cognitive deficits to language deficits, the *Boston Naming Test* (BNT, Goodglass et al., 1983), which was used to determine confrontation naming ability, the *Pyramids and Palm Trees* (PAPT, Howard and Patterson, 1992), which was used to test the participants’ semantic access, and the *American Speech-Language Hearing Association-Functional Assessment of Communication Skills for Adults* (ASHA-FACS, Frattali et al., 1995), which was used to determine the communication skills of the participants. Participants’ scores on the Aphasia Quotient (AQ) subtest from the WAB-R ranged from 11.5 to 99.9 (*M* = 68.9, *SD* = 26.6) and scores on the Composite Severity (CS) subtest from the CLQT ranged from 25 to 100% (*M* = 67.9, *SD* = 22.3). Participants’ scores on the BNT ranged from 0 to 98.3% (*M* = 47.7, *SD* = 37.4), scores on the PAPT ranged from 21.2 to 98.1% (*M* = 84.7, *SD* = 14.9), and scores on the Communication Independence (CI) mean score on the ASHA-FACS ranged from 3.2 to 6.98 out of 7 (*M* = 5.6, *SD* = 1.1) and the Qualitative Dimensions (QD) mean score ranged from 2.2 to 4.9 out of 5 (*M* = 3.8, *SD* = 0.8). Refer to **Table 1** for all participants’ standardized test scores.

### Stimuli

Thirty seven cognitive and linguistic treatment tasks, detailed in a Supplementary Table in the Des Roches et al. (2015) study, were implemented on an iPad using the Constant Therapy iOS platform. The tasks used a simple visual setup and similar methods of response were used across different tasks. For every task, instructions were provided in both a visual and auditory modality. Participants had the option of answering the item or skipping the item if they were unsure of the answer. Upon completion of each item, the application provided the correct answer and feedback on how the participants performed.

**FIGURE 2:**
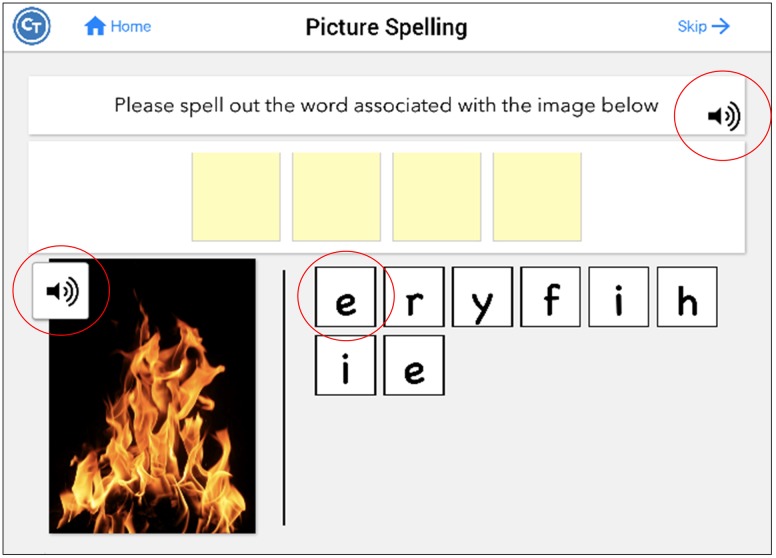
**Screenshot of the Picture Spelling task, which includes all possible hints**. Pressing the sound icon on the instruction bar repeats the instructions (repeat instructions). Pressing the sound icon on the picture plays the name of the picture (repeat audio stimulus). Pressing the letters plays the phonetic sound of the letters (play count). Figure obtained using Constant Therapy, www.constanttherapy.com.

Twenty-eight tasks included buttons that revealed a cue to answering the item. There were three types of auditory cues; the first repeated the instructions for completing the task (Repeat Instructions which repeated the instructions for completing the task), the second was the target stimulus presented auditorily (Repeat Audio Stimulus) (e.g., repeating the audio stimulus which was often necessary to complete the task), and the third was presentation of a phonemic cue or of a word (Play Count) (e.g., repeating additional audio stimuli provided within the task), which differed depending on the task. Refer to **Table 2** for a list of what self-administered cues were available by task. For example, in the Picture Spelling task participants were asked to determine the name of a picture and spell it from a bank of possible letters. In this particular task, all three types of cues were available to the participant (**Figure 2**): they can repeat the instructions (e.g., “Please spell out the word associated with the image below”); play the audio stimulus, which in this task, plays the name of the picture (e.g., fire); and play count, which plays the phonetic sound of the letters if pressed. Cues were self-administered so participants could use cues as often as they wanted. The software tracked every time a cue was utilized. It should be noted that these types of cues are typical in traditional speech-language therapy. While repetition of the stimulus upon request is often provided as a part of the treatment protocol, other types of cues mentioned above (e.g., initial phoneme, spoken word) have often been used in examinations of self-administered cues in treatment (Golper and Rau, 1983; Howard and Harding, 1998; Doesborgh et al., 2004; Tompkins et al., 2006).

**Table 2:** All tasks and corresponding cues provided in the treatment, including the task name, cognitive or language operation involved in completing the task, cue types (repeat instructions, repeat audio stimulus, or play count), a total number of cue types provided by task, and reason of exclusion for tasks not included in analyses.

Task	Cognitive/language operation	Repeat instructions	Repeat audio stimulus	Play count	Total # of cue types	Reason not included
Addition	Strengthening non-linguistic cognitive processing and selective working memory deficits	√			1	
Category identification	Distinguishing between semantically related and non-related words to strengthen semantic representations	√			1	
Clock math	Incrementally retraining quantitative reasoning skills by targeting linguistic cognitive processing, visuospatial, and working memory deficits	√			1	
Clock reading	Functionally strengthening visuospatial and spatial organization deficits via time judgment tasks	√			1	
Division	Strengthening non-linguistic cognitive processing and selective working memory deficits	√			1	
Instruction sequencing	Integrative reinforcement of goal directed executive functioning skills via functional planning and organization	√			1	
Long reading comprehension	Retraining sentence and story comprehension; literacy	√			1	
Map reading	Multimodal interventions to reinforce visuo-perceptual, scanning, and analytical reasoning skills	√			1	
Multiplication	Strengthening non-linguistic cognitive processing and selective working memory deficits	√			1	
Reading passage	Retraining sentence and story comprehension; literacy	√			1	
Subtraction	Strengthening non-linguistic cognitive processing and selective working memory deficits	√			1	
Word problem	Incrementally retraining quantitative reasoning skills by targeting linguistic cognitive processing, visual scanning, and working memory deficits	√			1	
Category matching	Semantically categorizing items to strengthen semantic representations	√	√		2	
Feature matching	Strengthening semantic representations	√	√		2	
Letter to sound matching	Retraining phoneme to grapheme conversion skills; develop sub-lexical analysis of words by identifying phonemes at the start/end of words	√		√	2	
Picture ordering	Multimodal intervention to improve task-related strategies while retraining analytical reasoning and working memory skills; retrieving phonological representations of words	√		√	2	
Rhyming	Retraining phonological encoding and processing	√	√		2	
Sound identification	Retraining phoneme processing	√	√		2	
Sound to letter matching	Retraining grapheme to phoneme conversion skills; develop sublexical analysis of words by identifying phonemes at the start/end of words	√	√		2	
Voice mail	Functionally reestablishing auditory working memory skills and task related strategies	√	√		2	
Word copy	Retraining visuospatial skills and orthographic representation in agraphia	√		√	2	
Word identification	Auditory word recognition	√	√		2	
Word ordering	Multimodal intervention to improve task-related strategies while retraining analytical reasoning and working memory skills	√		√	2	
Picture spelling	Retraining orthography via picture stimuli; phonological cueing, using phoneme to grapheme conversion	√	√	√	3	
Picture spelling completion	Retraining orthography via picture stimuli; phonological cueing, using phoneme to grapheme conversion	√	√	√	3	
Syllable identification	Retraining phonological segmentation	√	√	√	3	
Word spelling	Retraining orthography via auditory stimuli; phonological cueing, using phoneme to grapheme conversion	√	√	√	3	
Word spelling completion	Retraining orthography via auditory stimuli; phonological cueing, using phoneme to grapheme conversion	√	√	√	3	
Active sentence completion	Comprehension and production of canonical sentence structures				0	0 cues
Flanker	Response inhibition and mental flexibility; improving selective attention				0	0 cues
Naming picture	Retrieving semantic- phonological representations of words				0	0 cues
Passive sentence completion	Comprehension and production of non-canonical sentence structures				0	Only 1 session
Picture matching	Incrementally retraining visuospatial working memory				0	0 cues
Sound matching	Incrementally retraining auditory and spatial working memory				0	0 cues
Symbol matching	Systematically retraining visuospatial scanning and organization skills				0	0 cues
Word copy completion	Retraining visuospatial skills and orthographic representation in agraphia				0	Only 2 participants
Word matching	Incrementally retraining visuospatial working memory				0	0 cues

*√ = cue provided in the task*.

### Design and Procedures

The general design of the experiment is described in Kiran et al. (2014) and Des Roches et al. (2015). Briefly, participants’ language and cognitive profiles were assessed based on the standardized testing mentioned above. Each individual was then assigned several treatment tasks aimed at the targeted impairments indicated from the standardized assessments. Participants then practiced the assigned tasks during sessions in the clinic and at home for the 10 weeks treatment program, which was constantly monitored by clinicians to determine whether or not the participant’s schedule should be altered based on their performance. For example, if a participant were working on level three of Picture Spelling (which consists of seven or eight letter words) and achieved 97.5% accuracy one day and 99% accuracy on another day, the clinician would then change the level of difficulty for Picture Spelling to level four (which consists of eight or nine letter words). Therefore, real time monitoring and alteration of participants’ schedules resulted in an individualized treatment program for each participant that changed dynamically based on that participant’s performance over time [for more specific criteria, refer to Des Roches et al. (2015)]. The treatment design described in Des Roches et al. (2015) includes an experimental group and a control group, who were not different on their WAB-R AQ scores. The control group received 1 h of treatment once a week in the clinic, while the experimental group (*N* = 42) received the same clinic treatment but was also provided an iPad to take home and were encouraged to practice once each day for an hour. For the purposes of this study, these two groups are collapsed, as the effect of cue use on performance on the treatment tasks is not expected to differ based on the intensity of the treatment.

### Data Analysis

The Constant Therapy software generated reports for each participant, which included averaged accuracy for every session and a total count of all cue use in each session, specific to each level of all treatment tasks the participant completed. Any treatment tasks that did not provide any cues or that lacked enough data (see **Table 2**) were excluded, as well as any sessions where participants completed fewer than three items of a certain task.

To examine the relationship between self-administered cue use and accuracy (the first question), a *K*-means cluster analysis was completed for each participant (collapsing across time). A sum of squared error scree plot and the “pamk” function within the “fpc” package (Hennig, 2014) in “R” was run to determine how many clusters were appropriate for this dataset. Then, the cluster analysis was run for each participant to determine more closely what profile of relationship each individual showed between self-administered cue use and accuracy (cluster profile). To answer the second question, frequency of self-administered cue use was calculated for each participant by dividing the number of sessions in which the participant used one or more cue(s) by the total number of sessions the participant completed. Then, frequency of self-administered cues was correlated with scores on standardized tests and to determine how self-administered cue use and standardized test scores differed by these cluster profile, a MANOVA was performed. The above analyses collapsed trials across time, the different tasks, and across different types of cues; thus, these analyses examined the overall relationship of how self-administered cue use affected performance. To answer the third question, regressions were run to examine how self-administered cue use and time (treatment) affected accuracy for all participants and by participant. All analyses were completed in Statistical Package for the Social Sciences (SPSS Inc., Chicago, IL), Statistica software (StataCorp, College Station, TX, USA), and the statistical software package “R” (R Foundation for Statistical Computing, Vienna, Austria; R Core Team, 2014).

## Results

When examining the data collapsed over all participants and sessions, accuracy ranged from zero to 100% (*M* = 79.4, *SD* = 18.4) and total cue use ranged from zero to 199 cues (*M* = 8.6, *SD* = 18.9). Refer to **Table 1** for all participants’ percent cue use. Refer to the Supplementary Tables 1 and 2 for details about the specific tasks that each participant completed.

To determine how many clusters would be appropriate for a *K*-means cluster analysis, a sum of squared error scree plot of all participants’ data showed that either four or five clusters should be applied. Thus, the “pamk” function within the “fpc” package (Hennig, 2014) in “R” was run, which determined that five clusters was appropriate for the cluster analysis. If the initial result from the cluster analysis included one or more clusters with only one case, those cases were considered outliers, were deleted from the analysis, and the cluster analysis was run again until all clusters contained more than one case.

**FIGURE 3:**
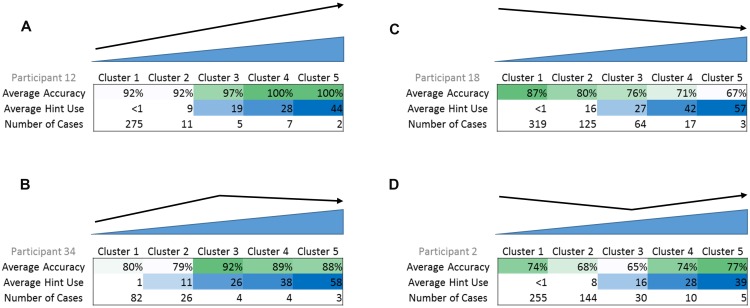
**Examples of the four cluster profiles, with average accuracy, average cue use, and number of cases in each cluster**. **(A)** Upward cluster profile, **(B)** curvilinear with initial upward trend cluster profile, **(C)** downward cluster profile, **(D)** curvilinear with initial downward trend cluster profile. In example **(A)**, as average cue use increases, average accuracy also increases. In example **(B)**, initially, as average cue use increases, average accuracy also increases. However, there is a peak and afterward, as average cue use increases, average accuracy begins to decrease.

Results from the cluster analyses indicated four different relationships or cluster profiles: (a) an increase in accuracy with greater self-administered cue use (upward, *N* = 13 participants), (b) a curvilinear trend with an initial increase in accuracy with greater self-administered cue use (curvilinear, initial upward, *N* = 9 participants), (c) a decrease in accuracy with greater self-administered cue use (downward, *N* = 11 participants), and (d) a curvilinear trend with an initial decrease in accuracy with greater self-administered cue use (curvilinear, initial downward, *N* = 14 participants) (see **Figure 3** for an example of cue use and accuracy cluster centers for each profile). All but four participants fit into these cluster profiles; the four participants who did not fit used little to no cues.

Next, to examine the potential relationship between self-administered cue use and severity of impairment based on standardized measures, a bivariate correlation was run to see how frequency of self-administered cue use (see **Table 1** for frequency of self-administered cue use by participant) correlated with pre-treatment scores on all of the standardized measures for all participants. Results showed that all measures negatively correlated with frequency of cue use where the greater the self-administered cue use, the lower the initial score on the standardized measure [WAB-R AQ: *r*(49) = -0.475, *p* < 0.01, CLQT CS: *r*(49) = -0.524, *p* < 0.001, BNT: *r*(49) = -0.517, *p* < 0.001, PAPT: *r*(48) = -0.500, *p* < 0.001, ASHA-FACS CI: *r*(44) = -0.641, *p* < 0.001, ASHA-FACS QD: *r*(44) = -0.603, *p* < 0.001]. All of these results survived a Bonferroni correction for multiple comparisons of *p* < 0.008.

**FIGURE 4:**
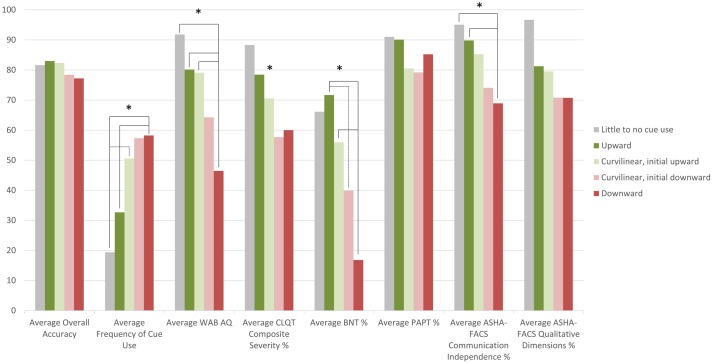
**Plot of participants’ average overall accuracy, average frequency of cue use and average standardized test scores by cluster profile**. For example, downward cluster profile participants have an overall average accuracy of 77.2%, an average frequency of 58.2 cues, an average WAB-R AQ score of 46.5, an average CLQT CS score of 60.0%, an average BNT score of 16.8%, an average PAPT score of 79.2%, an average ASHA-FACS CI score of 69.2%, and an average ASHA-FACS QD score of 70.8%. ^∗^Denotes the main effect was significant for that factor.

To further examine this relationship and to determine whether the greater self-administered cue use in more severe participants was beneficial to their performance, a one-way MANOVA was performed. The MANOVA examined if cluster profile (independent variable) had an effect on the different dependent variables (average overall accuracy throughout the treatment, frequency of self-administered cue use, and standardized measure scores at pre-treatment testing) (refer to **Figure 4** for the average scores of each dependent variable for each cluster profile). The MANOVA revealed that there was a significant difference in the dependent variables based on cluster profile, *F*(32,115.9) = 2.1, *p* = 0.003; Wilks’ Λ = 0.189, $\eta_{p}^{2}$ = 0.363. The main effect of cluster profile was significant for frequency of cue use [*F*(4,38) = 5.0, *p* = 0.002], WAB-R AQ scores [*F*(4,38) = 4.9, *p* = 0.003], CLQT CS scores [*F*(4,38) = 3.0, *p* = 0.03], BNT score [*F*(4,38) = 6.4, *p* < 0.001] and ASHA-FACS CI scores [*F*(4,38) = 4.8, *p* = 0.003], but not for average overall accuracy [*F*(4,38) = 1.3, *p* = 0.30], PAPT scores [*F*(4,38) = 1.4, *p* = 0.257], and ASHA-FACS QD scores [*F*(4,38) = 2.4, *p* = 0.06].

*Post hoc* Tukey honest significant difference (HSD) tests revealed differences between cluster profiles in several of the dependent measures. Frequency of cue use was significantly different across the cluster profiles; *downward* cluster profile participants (**Figure 3C**) use a significantly higher frequency of cues than participants in the *upward* cluster profile (**Figure 3A**, *p* = 0.01) and the little to no cue use profile (*p* = 0.04) and the *curvilinear with initial upward trend* cluster profile participants (**Figure 3B**) use a significantly higher frequency of cues (*p* = 0.03) than participants in the *upward* cluster profile. These results suggest that participants who used cues most frequently were also the ones that did not benefit from these cues, since they fell into the *downward* cluster profile. Conversely, participants who used a lower frequency of cues were also the ones that did benefit from these cues, since they fell into the *upward* or *little to no use* cluster profiles. WAB-R AQ scores were significantly different across cluster profiles; *downward* cluster profile participants had significantly lower AQ scores than the participants in the *upward* cluster profile (*p* = 0.005), participants in the *curvilinear with initial upward trend* cluster profile participants (*p* = 0.04), and participants in the *little to no cue use* cluster profile (*p* = 0.04) indicating that the participants who were most severe were also the ones who did not seem to benefit from self-administered cueing, and yet they used the most cues. The Tukey HSD *post hoc* analysis for CLQT CS scores showed no significant differences between the specific cluster profiles. BNT scores were significantly different across cluster profiles; *downward* cluster profile participants had significantly lower BNT scores than the participants in the *upward* cluster profile (*p* < 0.001) and the *curvilinear with initial upward trend* cluster profile participants (*p* = 0.03). Also, the *curvilinear with initial downward trend* cluster profile participants (**Figure 3D**) had significantly lower BNT scores than the participants in the *upward* cluster profile (*p* = 0.04), again indicating that the participants who were most severe were also the ones who did not seem to benefit from self-administered cueing. ASHA-FACS CI scores were significantly different across cluster profiles; *downward* cluster profile participants had significantly lower CI scores than the participants in the *upward* cluster profile (*p* = 0.01) and the *little to no cue use* cluster profile (*p* = 0.05).

To examine the third question, which looked at the relationship between cues and accuracy as a function of treatment, a regression was run collapsing across all participants. However, since the MANOVA showed differences in the relationship between accuracy and cue use by severity, participants were separated into two groups based on their initial severity (see **Table 1**). A WAB-R AQ score of 75 was the median score of this group, so participants who scored below 75 were included in the more severe bin (25 participants), while participants who scored 75 or above on the AQ were included in the less severe bin (26 participants). The two participants who could not complete the WAB-R were considered to fall in the more severe group determined by their performance in the treatment program and the remaining test scores.

Because this analysis included different levels of difficulty within each task over time, accuracy for each participant was normalized for levels within each task that comprised multiple levels (i.e., Word Spelling has five levels of the task). Therefore, if a participant achieved a raw accuracy score of 100% on Word Spelling level 1, they would receive a normalized accuracy score of 20%, while a raw accuracy score of 100% on Word Spelling level 5, they would receive a normalized accuracy score of 100%. In order to do this, each level within a task was assigned a progression order value in accordance with its difficulty. These values were used to calculate the normalized accuracy score for each session, using the following formula:

$$Normalized\,\;Score=\frac{\mathrm{Pr}ogression\,\;Order\,\;Value+Raw\,\;accuracy}{Number\,\;of\,\;total\,\;levels\,\;in\,\;task}$$

**FIGURE 5:**
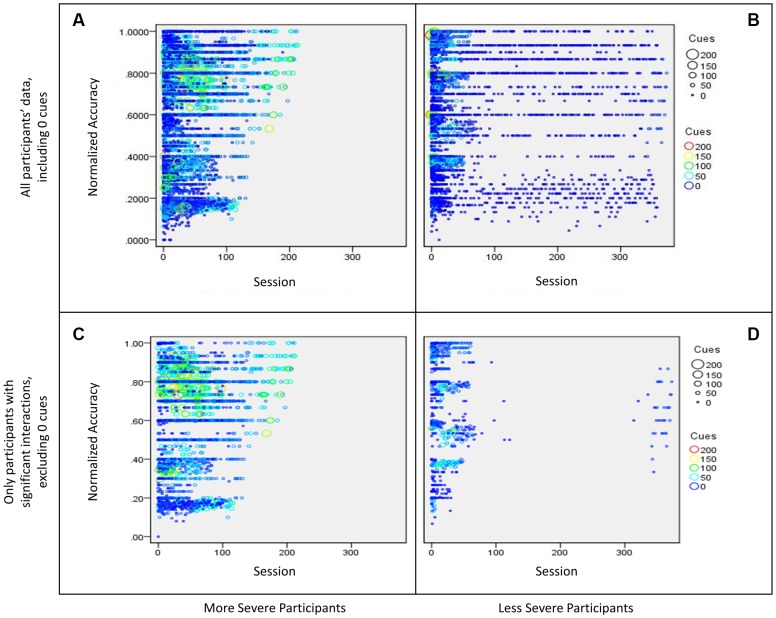
**Bubble plot of participants’ interaction between sessions (time) and cue use on normalized accuracy split by severity**. The size of the bubble as well as the color of the data point represents the number of cues used in a session. More specifically, **(A)** shows data for all more severe participants (WAB-R AQ < 75), including sessions where participants used zero cues, **(B)** shows data for all less severe participants (WAB-R AQ > 75), including sessions where participants used zero cues, **(C)** shows data for more severe participants that showed a significant interaction effect from the regression examining cue use and time on normalized accuracy, and **(D)** shows data for less severe participants that showed a significant interaction effect from the regression examining cue use and time on normalized accuracy.

Next, a regression was completed for the data of all 51 participants, which included a categorical variable (severity bin of more and less severe based on the AQ score) and two continuous variables (total cues and time, the latter was based on the number of times a participant completed a particular task) with normalized accuracy as the dependent measure. For the purpose of this paper, only the three-way interaction will be discussed because the effect of interest is how severity influences the effect of cue use on accuracy over the course of treatment. The three way interaction was significant [β = -0.03, *t*(13658) = 2.8, *p* = 0.006] and explained a significant proportion of variance in normalized accuracy scores [*R*^2^ = 0.02, *F*(7,13658) = 40.0, *p* < 0.01]. Results showed more severe participants used more cues than less severe participants, and while cue use decreased over time, the number of cues used per session was high (>50 cues) relative to the less severe participants as treatment sessions progressed. Additionally, these participants showed an increase in normalized accuracy over time (refer to panel A of **Figure 5**). The less severe participants (WAB-R AQ score above 75) showed heavy cue use initially, which decreased over time and participants eventually stopped using cues. Additionally, these participants show an increase in normalized accuracy over (refer to panel B of **Figure 5**). Once the dataset was graphed (see panels A and B of **Figure 5**), it was clear that the preponderance of sessions where participants used zero cues may have diluted the effects of using fewer cues vs. using many cues. To examine this issue, sessions with zero cues were excluded and the full regression was examined again. This time, the three-way interaction was not significant [β = -0.03, *t*(6033) = -0.48, *p* = 0.63, *f*^2^ = 0.06], however, as can be seen in panels C and D in **Figure 5**, the above mentioned pattern was more apparent. Specifically, more severe participants used more cues than less severe participants over time, and the number of cues used per session was high (>50 cues) relative to the less severe participants as treatment sessions progressed. Less severe participants used many cues initially in treatment but reduced their cue use over time. Both groups showed an increase in normalized accuracy over time.

In addition to the group level analysis, individual participant analyses reflected similar trends using the data where zero cues were excluded. Two-way interactions (cue use and time on normalized accuracy) are reported for each participant in **Table 3**. Briefly, of the 51 participants, 14 (27.5%) showed a significant two-way interaction; four participants showed a positive interaction effect while ten participants showed a negative interaction effect. In general, participants who showed positive effects tended to be less severe and participants who showed negative effects tended to be more severe. Notably, even though only 14 participants showed a significant effect, all but one of the participants showed at least a small effect size based on Cohen’s *f*^2^ (Cohen, 1988).

**Table 3:** Regression results for each participant, including the *R*^2^-value for the whole model and the β-value for the two-way interaction between time and cues, with the standard error in the parentheses.

ID	Whole model *R*^2^	Time^∗^Cues β	Cohen’s *f*^2^	Effect size
1	0.20	0.20 (0.31)	0.25552	Medium
2	0.02	-0.08 (0.11)	0.01939	Small
3	0.14^∗^	0.04 (0.14)	0.15819	Medium
4	0.21^∗∗∗^	-0.56 (0.13)^∗∗∗^	0.25796	Medium
5	0.31^∗∗∗^	-0.85 (0.25)^∗∗∗^	0.44541	Large
6	0.02	-0.04 (0.25)	0.01983	Small
7	0.57^∗∗∗^	-0.50 (0.16)^∗∗^	1.33005	Large
8	0.24^∗∗∗^	-0.45 (0.16)^∗∗^	0.32228	Medium
9	0.06	0.20 (0.23)	0.06128	Small
10	1	N/A	N/A	N/A
11	0.15^∗∗^	0.20 (0.18)	0.17299	Medium
12	0.15	-0.01 (0.23)	0.1816	Medium
13	0.43^∗∗∗^	-1.11 (0.25)^∗∗∗^	0.74574	Large
14	0.02	0.06 (0.15)	0.01728	Small
15	0.15^∗∗∗^	-0.08 (0.19)	0.17408	Medium
16	0.32^∗∗∗^	0.13 (0.30)	0.48106	Large
17	0.03	0.10 (0.23)	0.02729	Small
18	0.54^∗∗∗^	-0.14 (0.12)	1.15599	Large
19	0.45^∗∗^	-0.40 (0.28)	0.82777	Large
20	0.24^∗∗∗^	-0.69 (0.20)^∗∗∗^	0.31174	Medium
21	0.30^∗∗∗^	-0.40 (0.09)^∗∗∗^	0.43243	Large
22	0.43^∗∗∗^	0.21 (0.19)	0.76842	Large
23	0.50^∗∗∗^	0.62 (0.12)^∗∗∗^	1.01725	Large
24	0.05	-0.09 (0.23)	0.05742	Small
25	0.17^∗∗∗^	0.16 (0.13)	0.2093	Medium
26	0.18^∗∗∗^	-0.52 (0.09)^∗∗∗^	0.22327	Medium
27	0.17	-0.58 (0.36)	0.20674	Medium
28	0.51^∗∗∗^	0.41 (0.11)^∗∗∗^	1.02197	Large
29	0.59	-1.80 (1.12)	1.46612	Large
30	0.21	-0.03 (0.36)	0.26441	Medium
31	0.46^∗∗^	0.07 (0.22)	0.84766	Large
32	0.57	-0.12 (0.59)	1.35067	Large
33	0.11^∗∗∗^	0.09 (0.13)	0.12509	Small
34	0.04	0.09 (0.15)	0.04679	Small
35	0.16	-0.34 (0.44)	0.18549	Medium
36	0.24^∗∗∗^	0.22 (0.14)	0.31354	Medium
37	0.21	0.07 (0.44)	0.26852	Medium
38	0.14^∗∗^	0.33 (0.19)	0.16879	Medium
39	0.47	0.01 (0.57)	0.8849	Large
40	0.35^∗∗∗^	-0.10 (0.17)	0.53204	Large
41	0.16	0.19 (0.26)	0.18761	Medium
42	0.06	-0.12 (0.17)	0.06555	Small
43	0.27^∗∗^	0.73 (0.27)^∗∗^	0.37046	Large
44	0.30^∗∗∗^	-0.34 (0.16)^∗^	0.42317	Large
45	0.15^∗∗∗^	-0.69 (0.12)^∗∗∗^	0.17811	Medium
46	0.07	-0.14 (0.24)	0.07284	Small
47	0.09^∗∗∗^	0.24 (0.12)^∗^	0.10379	Small
48	0.14^∗∗∗^	0.07 (0.14)	0.16511	Medium
49	0.14	-0.47 (0.35)	0.16074	Medium
50	0.17	-0.28 (0.98)	0.20965	Medium
51	0.04	0.09 (0.26)	0.0454	Small

*^∗^Significant at the 0.05 probability level, ^∗∗^significant at the 0.01 probability level, ^∗∗∗^significant at the 0.001 probability level. Also included is the Cohen’s *f^*2*^*-value and the resulting effect size where an *f^*2*^* of 0.02 is considered to be a small effect, 0.15 a medium effect, and 0.35 a large effect.*

## Discussion

There are several important observations in this study. First, a variety of self-administered cue cluster profiles were found across a 51 participant sample, thus, not all participants used cues the same way. Four cluster profiles of relationships between cue use and accuracy were found from the cluster analyses, (a) higher accuracy with greater cue use, (b) curvilinear with initial high accuracy with greater cue use, (c) lower accuracy with greater cue use, and (d) curvilinear with an initial lower accuracy with greater cue use, with some additional participants showing little to no cue use throughout the treatment. Importantly, participants who fit the two curvilinear cluster profiles may have a threshold or pivot point at which cues became useful or not (**Figure 3**).

Second, a correlation that examined the overall relationship between severity of impairment based on standardized tests and frequency of cue use showed that more severe participants had a higher frequency of cue use. MANOVAs confirmed these findings indicating that participants who had a higher frequency of cue use, which was associated with lower accuracy, were the more severe participants in this sample. Conversely, participants who had a lower frequency of cue use, which was associated with higher accuracy, were the less severe participants in this sample.

Third, with regards to treatment, it was hypothesized that there would be several possible outcomes when considering the relationship between cue use and accuracy as a function of treatment. First, both groups of participants showed improved normalized accuracy over time. Interestingly, more severe participants used more cues than less severe participants over time, and the number of cues used per session was higher compared to the less severe participants as treatment sessions progressed. Less severe participants used many cues initially in treatment but reduced their cue use over time. These effects remained even after the data was culled to eliminate sessions where participants used zero cues. Also, though the two-way interaction may not have shown a significant effect for the majority of the participants, the variables included, time and cues used, significantly predicted normalized accuracy for almost all participants to differing levels.

These complex but important findings confirm the individual variability in the levels of assistance that participants need in shaping their behavior over the course of treatment. Each of these results will be discussed in some detail below.

When examining the cluster profiles of the relationship between accuracy and cue use for individual participants, results showed a general trend where the greater the cue use, the lower the participant’s accuracy, although some participants showed trends of higher accuracy with increased cue use. When examining the relationship between participants’ severity and self-administered cue use by cluster profile, participants in the upward cluster profile, who showed higher accuracy when using a higher number of cues in a session, scored significantly higher on standardized tests and had a significantly lower frequency of cue use. Therefore, the less severe participants tended to use a lower frequency of cues, but when cues were used, accuracy was higher. Additionally, participants in the downward cluster profile, who showed lower accuracy when using a higher number of cues in a session, scored significantly lower on standardized tests and had a significantly higher frequency of cue use. Therefore, the more severe participants tended to use a higher frequency of cues, which can be expected. However, contrary to the hypothesis, this greater use of cues was not beneficial to accuracy. Participants in the curvilinear cluster profiles tended to fall between the upward and downward cluster profile participants on both frequency of cue use and severity measures. Participants who used little to no cues scored significantly higher on many of the standardized test scores than more severe participants, demonstrating that participants who were the least impaired did not use cues very frequently.

As discussed in the introduction, it can be surmised that there are several important internal steps involved when one self-administers cues: the individual first needs to realize they do not know or cannot produce the correct answer, recognize that they need help in order to produce the correct answer, then self-administer the cue, and use the cue to help them produce the correct answer. To frame the current results in this context, in the case of less severe individuals, it may be possible that these individuals understand that they need the cue, self-administer the cue, and are able to more efficiently utilize the information provided by the cue. In contrast, in the case of severe individuals, these individuals understand that they need the cue and they self-administer the cue, however, the cues do not necessarily help them retrieve the correct answer. More severe individuals may also be reliant on cues as a compensatory or habitual routine which likely does not facilitate success. While the current study sheds some light on the first three steps entailed in self-administering a cue (as evidenced by the number and frequency of cue use), more work needs to be done to understand the fourth step, in terms of why some patients produce the correct answer and others do not. Nonetheless, information about an individual’s severity profile might help clinicians tailor treatment initially to provide the adequate number of cues for an individual. Importantly, a more severe individual might not benefit from further cueing initially, but might benefit from continued self-administered cue use as treatment continues.

To summarize, results addressing the first two research questions showed that increased use of cues was not always beneficial, particularly, the more severe individuals used the most cues and also tended to not benefit from these cues. While these results provided a cross-sectional snapshot of the data, the third research question examined the effect of cue-use over the course of treatment. The significant three way interaction of cues, time, and severity on normalized accuracy showed that more severe participants used more cues than less severe participants over time, and the number of cues used per session was higher compared to the less severe participants as treatment sessions progressed. Less severe participants used many cues initially in treatment but reduced their cue use over time. Both groups of participants showed improved normalized accuracy over time. Individual participant analyses completed as a follow up supported the group result. This effect might provide insight into tailoring the amount of cues provided throughout treatment. For instance, a less severe individual might not be likely to use cues, but should be encouraged to, since the use of cues is beneficial to them. Further, as the treatment progressed, these individuals may have learned to successfully perform the task without any cues. Conversely, a more severe individual might be more likely to use a lot of cues, and even though using more cues may not have an immediate beneficial effect on performance, cue use does indeed appear to support progression throughout treatment.

With additional research, it may be possible to structure a self-administered cue program, where cues are restricted based on severity profiles but are progressively altered throughout the treatment. One study (Ramsberger and Messamer, 2014) has used such a strategy of altering available cues but they did not systematically examine whether it was effective in contrast to all cues being available at all times. Previous research has also examined the effect of systematically decreasing vs. increasing cues throughout treatment. Results have shown similar findings for both decreasing and increasing cues (Conroy et al., 2009), although one study showed an effect only for increasing cues and also found that patients who were more severe showed a greater overall gain in treatment than patients who were less severe (Abel et al., 2005). Therefore, additional research is required to understand whether or not tailoring the cues available according to individual’s performance could be an effective treatment approach.

There are several factors to consider when understanding the implications of these findings. First, all levels of all tasks were collapsed for some analyses, which might lessen the effect of the results; not all tasks within each participant showed the same relationship between cue use and accuracy. Additionally, all cue types were collapsed; it is possible that not all cues would show similar relationships between cue use and accuracy across participants or even across tasks. For example, the repeat instructions cue in the Picture Spelling task provides information about what is expected of them in the task (“Please spell out the word associated with the image below”), whereas the same cue in the Rhyming task provides information that is necessary to answering the question (“Does [the word associated with the image below, e.g., “cherry”] rhyme with merry?). This differential effect of cue type with task might explain more about the relationship between cue use and accuracy. However, due to the individualized manner in which the treatment was administered, this type of analysis is beyond the scope of this study. Future research would be required to understand how the task might affect cue use and the resulting accuracies.

## Conclusion

To summarize, the results of the present study demonstrate that knowledge of an individual’s self-administered cue use is crucial for assessing and treating individuals with aphasia. It is possible that the use of self-administered cues helps some individuals to access information that is otherwise inaccessible. However, other individuals, who tended to be more severe, were not able to access the information with or without self-administered cues. Finally, as treatment progressed and accuracy increased, more severe participants used more cues than less severe participants over time, and the number of cues used per session was higher compared to the less severe participants as treatment sessions progressed. Less severe participants used many cues initially in treatment but reduced their cue use over time. Ultimately, the results demonstrate the need to monitor and individualize the levels of assistance available to individuals during rehabilitation.

## Ethics Statement

Boston University Institutional Review Board Informed consent was obtained prior to any measures or treatment for all participants, in accordance with policies set forth by the Boston University Institutional Review Board. The population involved in this study was people with aphasia. The individuals’ ability to understand and give informed consent was assessed by Dr. Swathi Kiran, who has over 10 years in experience evaluating and treatment of peoples with aphasia. Individuals’ spouses and next of kin (if applicable) were also be involved in the consent process. During this meeting, the potential benefits and risks of the experiment were explained to the individual and their family (surrogates).

## Author Contributions

CD contributed to the acquisition and interpretation of the data for the work and contributed greatly to drafting and revising the work. AM contributed to the interpretation of the data for the work and contributed to revising the work. SK contributed to the concept, design of, and interpretation of data for the work, and also contributed to revising the work.

## Conflict of Interest Statement

There is a significant financial relationship. Boston University owns a portion of stock equity in Constant Therapy, the software company that delivered the treatment. CD owns a portion of the stock equity that BU owns. SK is the co-founder and Scientific Advisor in Constant Therapy and owns stock equity in Constant Therapy. The results of the study are independent of the software platform and therefore there is no scientific overlap. The other author declares that the research was conducted in the absence of any commercial or financial relationships that could be construed as a potential conflict of interest.
