# Supplementary material for: Relationship between Self-Administered Cues and Rehabilitation Outcomes in Individuals with Aphasia: Understanding Individual Responsiveness to a Technology-Based Rehabilitation Program
Source: Front Hum Neurosci. 2017 Feb 1;11:07. doi: 10.3389/fnhum.2017.00007 (PMC5285333; doi:10.3389/fnhum.2017.00007)
Supplement: Supplementary file 1 [file Table_1.PDF]

# *Supplementary Material*

# Relationship between level of assistance and rehabilitation outcomes in individuals with aphasia: Understanding individual responsiveness to a technology-based rehabilitation program

**Carrie Des Roches*, Annette Mitko, Yorghos Tripodis, & Swathi Kiran**

*** Correspondence:** Carrie Des Roches: cadesroc@gmail.com

## 1 Supplementary Table

**Supplementary Table 1. Table of language tasks completed by each participant.** Tasks highlighted in gray were completed by the participant; cells with * denote tasks only completed once.

| | Language Tasks | | | | | | | | | | | | | | | |
|---|---|---|---|---|---|---|---|---|---|---|---|---|---|---|---|---|
| | Naming | | | | | Reading | | | | | | Writing | | | | |
| ID | Category Matching | Feature Matching | Rhyming | Sound Identification | Syllable Identification | Category Identification | Letter to Sound Matching | Sound to Letter Matching | Reading Passage | Long Reading Comprehension | Word Identification | Word Copy | Word Spelling | Word Spelling Completion | Picture Spelling | Picture Spelling Completion |
| 1 | | * | | | | | | | | | * | * | * | | | |
| 2 | | | | | | | * | | | | * | | | | | |
| 3 | | | | | | | * | | | | | | | | | |
| 4 | * | | * | | | | * | * | * | | | * | | | | |
| 5 | | | | | | | | | | | | | | | | |
| 6 | | | | | | * | | | | | | * | | | | |
| 7 | | | | | | | | | | | * | * | | | | |
| 8 | * | * | | | | * | | * | | | * | | | | | |

| ID | Language Tasks | | | | | | | | | | | | | | | |
|---|---|---|---|---|---|---|---|---|---|---|---|---|---|---|---|---|
| | Naming | | | | | Reading | | | | | | Writing | | | | |
| | Category Matching | Feature Matching | Rhyming | Sound Identification | Syllable Identification | Category Identification | Letter to Sound Matching | Sound to Letter Matching | Reading Passage | Long Reading Comprehension | Word Identification | Word Copy | Word Spelling | Word Spelling Completion | Picture Spelling | Picture Spelling Completion |
| 9 | | | * | | * | | | | | | | | | | * | |
| 10 | | | | | | | | | | | | | | | | |
| 11 | * | * | | * | | * | | | | | * | | | * | | |
| 12 | * | * | * | * | | * | | | | | | | * | | | |
| 13 | | | | | | | | | * | | * | | | | | |
| 14 | * | * | * | * | | | | | | | | | | | | |
| 15 | | * | | | * | | * | * | | | * | * | | | | |
| 16 | * | | | | | * | | | | | | | | | | |
| 17 | | | | | | | | | | | * | | | | | |
| 18 | | | | | | * | | | | | | | | | | |
| 19 | | | | | | | | | | | | | | | * | |
| 20 | | | | | | | | | | | | | | | | |
| 21 | | | | | | | | | | | | | | | | |
| 22 | | | | | | | | | | | | * | * | | | |
| 23 | | | | | | | | | | | * | | | | | |
| 24 | | | | | | | * | | | | | | | | | |
| 25 | | | | | | * | | | | | | | | | | |
| 26 | | * | | | * | | | | | | | | | | | |
| 27 | | | | | | | | | | | | | | | | |
| 28 | * | | | | | | | * | | | | | | | | |
| 29 | | | | | | | | | * | * | | | | | | |
| 30 | | | | | | | | | | | | | | | | |

| ID | Language Tasks | | | | | | | | | | | | | | | |
|---|---|---|---|---|---|---|---|---|---|---|---|---|---|---|---|---|
| | Naming | | | | | Reading | | | | | | Writing | | | | |
| | Category Matching | Feature Matching | Rhyming | Sound Identification | Syllable Identification | Category Identification | Letter to Sound Matching | Sound to Letter Matching | Reading Passage | Long Reading Comprehension | Word Identification | Word Copy | Word Spelling | Word Spelling Completion | Picture Spelling | Picture Spelling Completion |
| 31 | | | | | | | | | | | | | | | | |
| 32 | | | | | | | | | | | | | | | * | |
| 33 | | | | | | | | | | | * | | | | | |
| 34 | | | | * | * | | * | * | | | | | | | | |
| 35 | | | | | | | | | | | | | | | | |
| 36 | * | * | * | | | | | * | | | | | | | * | |
| 37 | | | | | | | * | | | | * | | | | | |
| 38 | * | | | * | * | | | | | | | | | | | |
| 39 | | | | | | | | | | | | | | | | |
| 40 | | | | | | | | | | | | | | | | |
| 41 | | | | | | | | | | | | | | | | |
| 42 | | | | | * | | | | | | | | * | | | |
| 43 | | | | * | | | | | | | | | | | | |
| 44 | | | | | | | * | | | | | | | | | |
| 45 | | | | | | | | | | | | | | | | |
| 46 | | | | | | | | | | | | | | | | |
| 47 | | | | | | | | | * | | | | | | | |
| 48 | | | | | | * | | | | | | * | | | | |
| 49 | | | * | | | | | | | | | | | | | |
| 50 | | | | | | | | | | | | | | | | |
| 51 | | | | | | | | | | | | | | * | | |
