# Supplementary material for: Relationship between Self-Administered Cues and Rehabilitation Outcomes in Individuals with Aphasia: Understanding Individual Responsiveness to a Technology-Based Rehabilitation Program
Source: Front Hum Neurosci. 2017 Feb 1;11:07. doi: 10.3389/fnhum.2017.00007 (PMC5285333; doi:10.3389/fnhum.2017.00007)
Supplement: Supplementary file 2 [file Table_2.PDF]

Supplementary Material

Relationship between level of assistance and rehabilitation outcomes in individuals with aphasia: Understanding individual responsiveness to a technology-based rehabilitation program

Carrie Des Roches*, Annette Mitko, Yorghos Tripodis, & Swathi Kiran

*** Correspondence:** Carrie Des Roches: cadesroc@gmail.com

1 Supplementary Table

Supplementary Table 2. Table of cognitive tasks completed by each participant. Tasks highlighted in gray were completed by the participant; cells with * denote tasks only completed once.

	Cognitive Tasks											
	Auditory Memory	Visual Processing	Analytical Reasoning			Arithmetic				Quantitative Reasoning		Executive Skills
ID	Voice Mail	Clock Reading	Map Reading	Picture Ordering	Word Ordering	Addition	Subtraction	Multiplication	Division	Clock Math	Word Problem	Instruction Sequencing
1					*							
2			*			*						
3												
4					*							
5												
6					*							
7				*		*						
8		*										
9	*											
10												

ID	Cognitive Tasks											
	Auditory Memory	Visual Processing	Analytical Reasoning			Arithmetic				Quantitative Reasoning		Executive Skills
	Voice Mail	Clock Reading	Map Reading	Picture Ordering	Word Ordering	Addition	Subtraction	Multiplication	Division	Clock Math	Word Problem	Instruction Sequencing
11					*							
12		*										
13							*					
14												
15					*				*			
16												
17												
18												
19						*						
20												
21												
22								*				
23												
24												
25		*										
26												
27												
28			*									
29	*	*						*				
30												
31		*										
32	*								*			
33												
34		*			*							*

	Cognitive Tasks											
	Auditory Memory	Visual Processing	Analytical Reasoning			Arithmetic				Quantitative Reasoning		Executive Skills
ID	Voice Mail	Clock Reading	Map Reading	Picture Ordering	Word Ordering	Addition	Subtraction	Multiplication	Division	Clock Math	Word Problem	Instruction Sequencing
35												*
36												
37		*										
38												
39		*			*	*	*					
40												
41												
42		*			*							*
43												
44		*										
45												
46												*
47												
48		*										
49							*					
50						*						
51												
